# Identification and Validation of Immune Infiltration Phenotypes in Laryngeal Squamous Cell Carcinoma by Integrative Multi-Omics Analysis

**DOI:** 10.3389/fimmu.2022.843467

**Published:** 2022-02-24

**Authors:** Li Yan, Xiaole Song, Gang Yang, Lifen Zou, Yi Zhu, Xiaoshen Wang

**Affiliations:** ^1^ Department of Radiation Oncology, Eye & ENT Hospital, Fudan University, Shanghai, China; ^2^ Department of Otolaryngology, Eye & ENT Hospital, Fudan University, Shanghai, China

**Keywords:** head and neck cancer, laryngeal, immunology, TCGA, immune infiltration

## Abstract

**Background:**

Laryngeal squamous cell carcinoma (LSCC) is one of the world’s most common head and neck cancer. However, the immune infiltration phenotypes of LSCC have not been well investigated.

**Methods:**

The multi-omics data of LSCC were obtained from the TCGA (n=111) and GEO (n=57) datasets. The infiltrations of the 24 immune cell populations were calculated using the GSVA method. Then LSCC samples with different immune cell infiltrating patterns were clustered, and the multi-omics differences were investigated.

**Results:**

Patients were clustered into the high-infiltration and low-infiltration groups. The infiltration scores of most immune cells were higher in the high-infiltration group. Patients with high-infiltration phenotype have high N and TNM stages but better survival, as well as less mutated COL11A1 and MUC17. Common targets of immunotherapies such as PD1, PDL1, LAG3, and CTLA4 were significantly up-regulated in the high-infiltration group. The differentially expressed genes were mainly enriched in several immune-related GOs and KEGG pathways. Based on the genes, miRNAs, and lncRNAs differentially expressed in both the TCGA and GEO cohorts, we built a ceRNA network, in which BTN3A1, CCR1, miR-149-5p, and so on, located at the center. A predictive model was also constructed to calculate a patient’s immune infiltration phenotype using 16 genes’ expression values, showing excellent accuracy and specificity in the TCGA and GEO cohorts.

**Conclusions:**

In this study, the immune infiltration phenotypes of LSCC and the corresponding multi-omics differences were explored. Our model might be valuable to predicting immunotherapy’s outcome.

## Introduction

As one of the most common head and neck cancers worldwide, laryngeal carcinoma accounts for 184,615 new cases and 99,840 deaths in 2020, of which >95% are laryngeal squamous cell carcinomas (LSCC) (1). The early-stage LSCC patients have favorable treatment effects through radical surgery or radiation (2, 3). However, nearly half of the patients present with advanced disease at first diagnosis, for whom surgery combined radiation and chemotherapy is the current standard treatment, facing the challenge of effective local disease control and preservation of laryngeal function (4, 5). The 5-year overall survival (OS) for LSCC patients is approximately 63%, which changed little over past years, without outstanding advancement in both diagnosis and treatment (4).

At present, immunotherapy is becoming one of the most promising modes for various tumors, including LSCC (6). Immunotherapy, such as blocking the interaction between PD-1 and its ligands, boosts immunity and greatly affects both radiotherapy and chemotherapy for LSCC (6). The immune-infiltration landscape may act as immunotherapeutic biomarkers and potential therapeutic targets for LSCC, but it’s still not well explored (7).

In the present study, we comprehensively analyzed the immune infiltration phenotypes of LSCC and the correlated clinical parameters. The molecular characteristics of different infiltration phenotypes were also investigated and validated using multi-omics data, including mutation, and the expression of genes, miRNAs, and lncRNAs. We hope that our research will help improve the understanding of the immune status and provide new prospects for the immunotherapy of LSCC.

## Materials and Methods

### Data Source and Processing

Log2(FPKM+1) gene expression RNA-seq data, somatic mutation (MuTect2) data, copy number (gene-level) data, and log2(RPM+1) miRNA mature strand expression RNA-seq Data, of Head and Neck Cancer samples of The Cancer Genome Atlas (TCGA), were downloaded from the UCSC Xena browser (https://gdc.xenahubs.net) as well as the corresponding clinical and survival information (8–10). The inclusion criteria for LSCC patients in the TCGA database were the patients whose sites of rection were larynx. Normal samples, recurrent tumor samples, or paraffin-embedded samples were excluded. We have added the inclusion and exclusion criteria in the method section as suggested. RNA-Seq data of GSE127165 and miRNA-Seq data of GSE133632 in the GEO database (https://www.ncbi.nlm.nih.gov/gds/) were used as the validation cohorts, and the data were also log2 conversed before being analyzed (11).

### Calculation and Clustering of Immune Cell Infiltration

As previously reported (12–14), the expression profile of 585 immune cell infiltration related genes was used to calculate the infiltrations of the 24 immune cell populations based on the Gene Set Variation Analysis (GSVA) method using R software (version 4.0.5). A matrix containing the infiltration enrichment scores ranging from -1 to 1 for each immune cell type in every tumor sample was obtained. Then tumor samples with different immune cell infiltrating patterns were clustered and grouped by an unsupervised clustering method.

### Clinical and Survival Analyses

The clinical characteristics of the immune infiltration groups were compared using t-test, Wilcoxon, and chi-square tests. Kaplan-Meier method, log-rank test, univariate and multivariate cox analyses were used to investigate the survival. All the statistical analyses here were performed using R.

### Comparison of Somatic Mutations, Copy Number Variation, the Expression of Genes, MiRNAs, and LncRNAs

Somatic mutations and copy number variation were analyzed using the maftools of R. The threshold of *p*-value < 0.01 was considered significant. Differentially expressed genes (DEGs), miRNAs, and lncRNAs were identified in different immune infiltration phenotypes using the package limma of R, with a threshold of Log (fold change) > 0.5, *p*-value < 0.05, and adjusted *p*-value < 0.05.

### Functional Analyses of DEGs and Constructions of CeRNA Network

Functional enrichment analyses of Gene Ontology (GO) and Kyoto Encyclopedia of Genes and Genomes (KEGG) were performed with the clusterProfiler package of R, with a cutoff of adjusted P < 0.01 and false discovery rate (FDR) < 0.05. ceRNA network was built and visualized by Cytoscape (version 3.7.1) to further explore the relationship among DEGs, the differentially expressed miRNAs and lncRNAs. The regulations in the ceRNA network were predicted by miRwalk and miRcode. Only the targets which were down-regulated when the miRNAs were up-regulated were included and vice versa.

### Construction of the Prediction Model

The LASSO regression was used in this study to select the optimal set of genes to calculate to which immune infiltration phenotype a patient belongs. Ten-fold cross-validation was adopted using the glmnet package in R to determine the optimal parameter λ and the corresponding set of genes. Then binary logistic regression was applied to construct the model using the expression of selected genes. At the same time, the ROC curve was used to determine the best cutoff value of the model and calculate its accuracy and specificity.

## Results

### Classification of the Immune Infiltration Phenotypes of LSCC Patients in the TCGA Database

**Figure 1:**
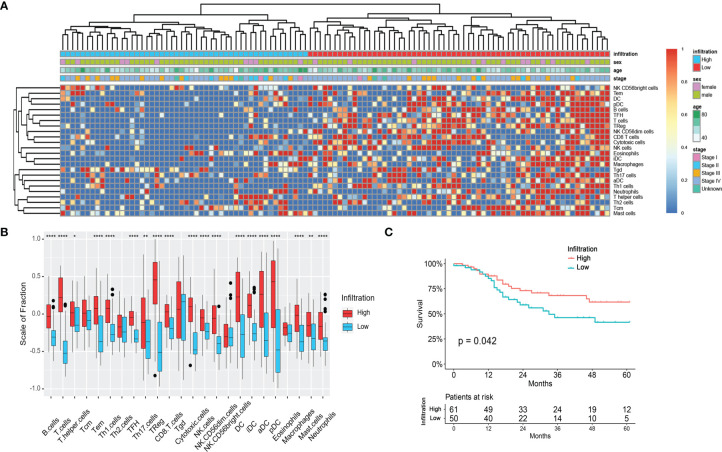
Immune infiltration status of the LSCC patients in the TCGA cohort. **(A)** Unsupervised clustering of 24 types of immune cells for 111 LSCC patients. **(B)** Fraction of immune cells in LSCC patients with high-infiltration and low-infiltration phenotypes. *p < 0.05; **p < 0.01; ****p < 0.0001. **(C)** Survival analysis of LSCC patients with high-infiltration and low-infiltration phenotypes.

A total of 111 LSCC patients in the TCGA head and neck cancer cohort were enrolled in this study. Using the GSVA enrichment method, the infiltration of each immunity cells in each patient was calculated and then clustered into two different groups (high-infiltration group, n = 61, and low-infiltration group, n = 50) (**Figure 1A**). The infiltration scores of 21/24 immune cells were higher in the high-infiltration group than those in the low-infiltration group (**Figure 1B**).

The clinical characteristics were compared between the two immune infiltration phenotypes. As shown in **Table 1** and **Figure 1A**, the N stage of the patients in the high-infiltration group was significantly lower (*p* = 0.048) as well as the TNM stage (*p* = 0.025), while there was no significant difference between the two infiltration groups regarding race, sex, age, grade, T stage, M stage, smoking or alcohol status. The 1-year, 3-year, and 5-year survival of patients with high-infiltration phenotype were 87.7 ± 4.4%, 68.0 ± 6.7%, and 61.5 ± 7.5%, respectively, significantly better than those of patients with low-infiltration (85.2 ± 5.2%, 46.1 ± 8.2%, 41.0 ± 8.7%, respectively) (*p* = 0.042) (**Figure 1C**).

**Table 1:** Clinical characteristics of patients with different immune infiltration phenotypes.

Characteristics	Total	High-Infiltration	Low-Infiltration	*p* value
**No.**	111 (100%)	61 (55.0%)	50 (45.0%)	
**Race**				0.645
White	86 (77.5%)	46 (75.4%)	40 (80.0%)	
Black	19 (17.1%)	12 (19.7%)	7 (14.0%)	
Others	6 (5.4%)	3 (4.9%)	3 (6.0%)	
**Sex**				
Male	92 (82.9%)	51 (83.6%)	41 (82.0%)	0.823
Female	19 (17.1%)	10 (16.4%)	9 (18.0%)	
**Age (years old)**	61.8 ± 9.3	61.6 ± 8.4	62.1 ± 10.2	0.750
Grade				0.840
G1	8 (7.2%)	5 (8.2%)	3 (6.0%)	
G2	70 (63.1%)	37 (60.7%)	33 (66.0%)	
G3	29 (26.1%)	17 (27.9%)	12 (24.0%)	
Gx	4 (3.6%)	2 (3.3%)	2 (4.0%)	
**Stage**				0.025
I	3 (2.7%)	1 (1.6%)	2 (4.0%)	
II	11 (9.9%)	3 (4.9%)	8 (16.0%)	
III	26 (23.4%)	13 (21.3%)	13 (26.0%)	
IV	67 (60.4%)	42 (68.9%)	25 (50.0%)	
Unknown	4 (3.6%)	2 (3.3%)	2 (4.0%)	
**T stage**				0.204
T1	3 (2.7%)	1 (1.6%)	2 (4.0%)	
T2	17 (15.3%)	7 (11.5%)	10 (20.0%)	
T3	35 (31.5%)	20 (32.8%)	15 (30.0%)	
T4	52 (46.8%)	31 (50.8%)	21 (42.0%)	
Tx	4 (3.6%)	2 (3.3%)	2 (4.0%)	
**N stage**				0.048
N0	55 (49.5%)	25 (41%)	30 (60.0%)	
N1	18 (16.2%)	12 (19.7%)	6 (12.0%)	
N2	29 (26.1%)	19 (31.1%)	10 (20.0%)	
N3	3 (2.7%)	2 (3.3%)	1 (2.0%)	
Nx	6 (5.4%)	3 (4.9%)	3 (6.0%)	
**M stage**				1.000
M0	104 (93.7%)	58 (95.1%)	46 (92.0%)	
M1	2 (1.8%)	1 (1.6%)	1 (2.0%)	
Mx	5 (4.5%)	2 (3.3%)	3 (6.0%)	
**Smoking Status**				0.855
No	17 (15.3%)	10 (16.4%)	7 (14.0%)	
Yes	91 (82.0%)	49 (80.3%)	42 (84.0%)	
Unknown	3 (2.7%)	2 (3.3%)	1 (2.0%)	
**Alcohol Status**				0.851
No	39 (35.1%)	21 (34.4%)	18 (36.0%)	
Yes	70 (63.1%)	39 (63.9%)	31 (62.0%)	
Unknown	2 (1.8%)	1 (1.6%)	1 (2.0%)	

### Gene Mutations Associated With Immune Infiltration Phenotypes of LSCC Patients in the TCGA Database

**Figure 2:**
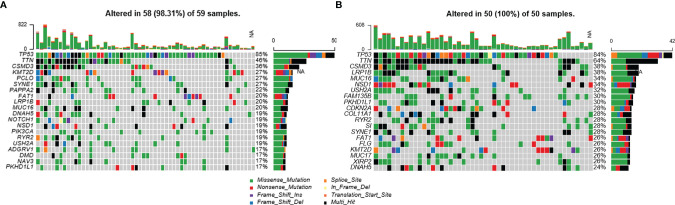
Mutation status of LSCC patients with different immune-infiltration phenotypes in the TCGA cohort. **(A)** Patients with high-infiltration phenotype. **(B)** Patients with low-infiltration phenotype. NA, Not Available.

One hundred and nine LSCC patients with gene mutation data were included in this analysis. As **Figure 2** shows, the gene mutation status was quite different between patients with different immune infiltration phenotypes. There was a median of 120 and 161.5 mutations in each patient with a high-infiltration phenotype and a low-infiltration group, respectively. The most common DNA base mutation type was C>T in the high-infiltration group, but it was C>A in the low infiltration group (**Supplementary Figure 1**).

The most commonly mutated genes in the high-infiltration group were TP53 (85%), TTN (46%), CSMD3 (36%), KMT2D (27%), and PCLO (27%), while in the low-infiltration group they were TP53 (84%), TTN (64%), CSMD3 (38%), and MUC16 (34%). In the top 20 mutated genes of either group, COL11A1 (7% vs. 28%, *p* = 0.004), MUC17 (8% vs. 26%, *p* = 0.019), FAM135B (12% vs. 30%, *p* = 0.030), and XIRP2 (10% vs.26%, *p* = 0.042) were significantly less mutated in the high-infiltration group (**Figure 2** and **Supplementary Table 1**). These differentially mutated genes might contribute to the distinct immune infiltration phenotypes.

### Differently Expressed Genes, MiRNAs, and LncRNAs Analyses Between Different Immune Infiltration Phenotypes of LSCC Patients in the TCGA Database

**Figure 3:**
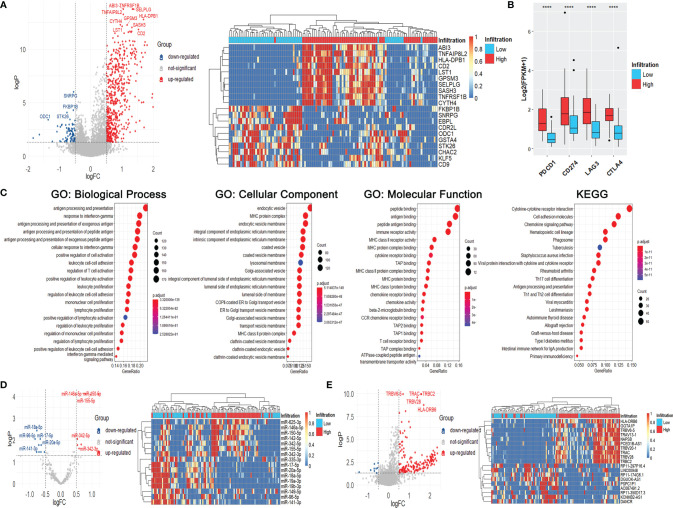
Immune infiltration status, differentially expressed genes, miRNAs, and lncRNAs of LSCC patients with different immune-infiltration phenotypes in the TCGA cohort. **(A)** Volcano plot and heatmap of DEGs. **(B)** Expression of common targets of immune therapies. ****p < 0.0001. **(C)** GO and KEGG analyses of DEGs. **(D)** Volcano plot and heatmap of differentially expressed miRNAs. **(E)** Volcano plot and heatmap of differentially expressed lncRNAs.

DEGs between the high-infiltration and low-infiltration groups were firstly investigated using the TCGA data and further analyzed to identify the potential biological functions. As **Figure 3A** shows, a total of 558 up-regulated and 82 down-regulated DEGs were identified in the high-infiltration group compared with the low-infiltration group. The top-3 up-regulated genes with the lowest *p*-values were TNFRSF1B (logFC = 1.33), ABI3 (logFC = 1.04), and SELPLG (logFC = 1.36) (all *p* < 0.001), while the top-3 down-regulated genes were SNRPG (logFC = -0.55), FKBP1B (logFC = -0.56), and STK26 (logFC = -0.76) (all *p* < 0.001) (**Figure 3A**). Meanwhile, four targets of immunotherapies, PDCD1 (PD1), CD274 (PD-L1), LAG3, and CTLA4, were all significantly ug-regulated in patients with high immune infiltration phenotype, indiciting those patients might be more suitable for immunotherapy (**Figure 3B**).

The results of GO and KEGG analyses showed that the DEGs were significantly enriched in immune-related gene ontologies and pathways (**Figure 3C**), indicating the huge divergence between the two immune phenotypes.

Meanwhile, seven ug-regulated and nine down-regulated miRNAs were identified, including miR-150-5p (logFC = 1.21), miR-146a-5p (logFC = 0.80), miR-18a-5p (logFC = -0.77) and miR-96-5p (logFC = -0.83) (all *p* < 0.001) (**Figure 3D**). One hundred and eighty-three up-regulated and nine down-regulated lncRNAs were also identified, including TRBC2 (logFC = 1.57), TRAC (logFC = 1.56), RP11-174G6.1 (logFC = -0.50), and DANCR (logFC = -0.69) (all *p* < 0.001) (**Figure 3E**).

### Validation of Differently Expressed Genes, MiRNAs, and LncRNAs Using GEO Data

We searched the GEO database and chose GSE127165 and GSE133632, RNA-Seq and miRNA-Seq data of the same 57 LSCC patients, the largest cohort in the GEO database, as the validation cohort. There is a lack of clinical, survival, or gene mutation information in the GEO dataset. Therefore, unfortunately, we couldn’t validate the results of clinical, survival, and gene mutation analyses in the GEO data.

**Figure 4:**
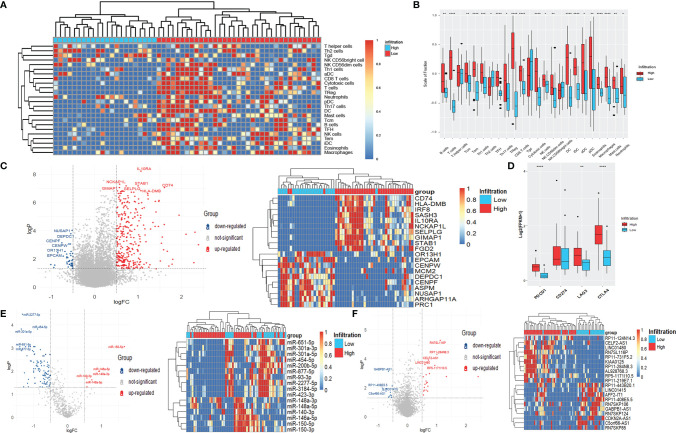
Differentially expressed genes, miRNAs, and lncRNAs of LSCC patients with different immune-infiltration phenotypes in the GEO cohort. **(A)** Unsupervised clustering of 24 types of immune cells for 57 LSCC patients. **(B)** Fraction of immune cells in LSCC patients with high-infiltration and low-infiltration phenotypes. *p < 0.05; **p < 0.01; ***p < 0.001; ****p < 0.0001. **(C)** Volcano plot and heatmap of DEGs. **(D)** Expression of common targets of immune therapies. **p < 0.01; ****p < 0.0001. **(E)** Volcano plot and heatmap of differentially expressed miRNAs. **(F)** Volcano plot and heatmap of differentially expressed lncRNAs.

The 57 patients were also clustered into high-infiltration (n = 35) and low-infiltration (n = 22) groups (**Figure 4A**). The infiltration scores of most immune cells were higher in the high-infiltration group than those in the low-infiltration group, consistent with the results of the TCGA analyses (**Figure 4B**). As **Figure 4B** shows, 309 up-regulated DEGs and 43 down-regulated DEGs were indetified, including IL10RA (logFC = 1.06), NCKAP1L (logFC = 0.57), STAB1 (logFC = 1.00), NUSAP1 (logFC = -0.52), DEPDC1 (logFC = -0.55), and CENPF (logFC = -0.76) (all *p* < 0.001) (**Figure 4C**). The expression of PDCD1, LAG3, and CTLA4 were significantly raised in high-infiltration group, too (**Figure 4D**). The DEGs were also siginicantly enriched in lots of immue-related gene ontologies and pathways (**Supplementary Figure 2**), quite similar as the GO and KEGG results of the TCGA data.

Six up-regulated miRNAs, including miR-150-5p (logFC = 1.68), miR-146a-5p (logFC = 0.94), and miR-148a-3p (logFC = 0.92), as well as 108 down-regulated miRNAs, including miR-2277-5p (logFC = -1.33), miR-301a-5p (logFC= -1.36), and miR-454-5p (logFC = -0.88) (all p *<* 0.001) were identified (**Figure 4E**). Meanwhile, 21 up-regulated lncRNAs and 12 down-regulated lncRNAs, including RN7SL116P (logFC = 1.11), RP11-284N8.3 (logFC = 1.00), GABPB1-AS1 (logFC = -0.65), and RP11-443B20.1 (logFC = -0.52) (all p < 0.001) were significantly differentially expressed between the two immune infiltration phenotypes (**Figure 4F**).

The intersections of differentially expressed genes, miRNAs, and lncRNAs obtained from the TCGA and GEO data were analyzed. As **Supplementary Figures 3A**, **B** shows, 202 genes (197 up-regulated and five down-regulated) and eight miRNAs (two up-regulated and six down-regulated) were differentially expressed both in the TCGA and GEO data, accounting for a large part of obtained DEGs and differentially expressed miRNAs, which demonstrates that the classification of immune infiltration phenotypes in our study is reasonable and universal. However, there was only one lncRNA, AL928768.3, differentially expressed in both two data cohorts (**Supplementary Figure 3C**), possibly due to the low and unstable expression of lncRNAs.

### Construction of CeRNA Network

**Figure 5:**
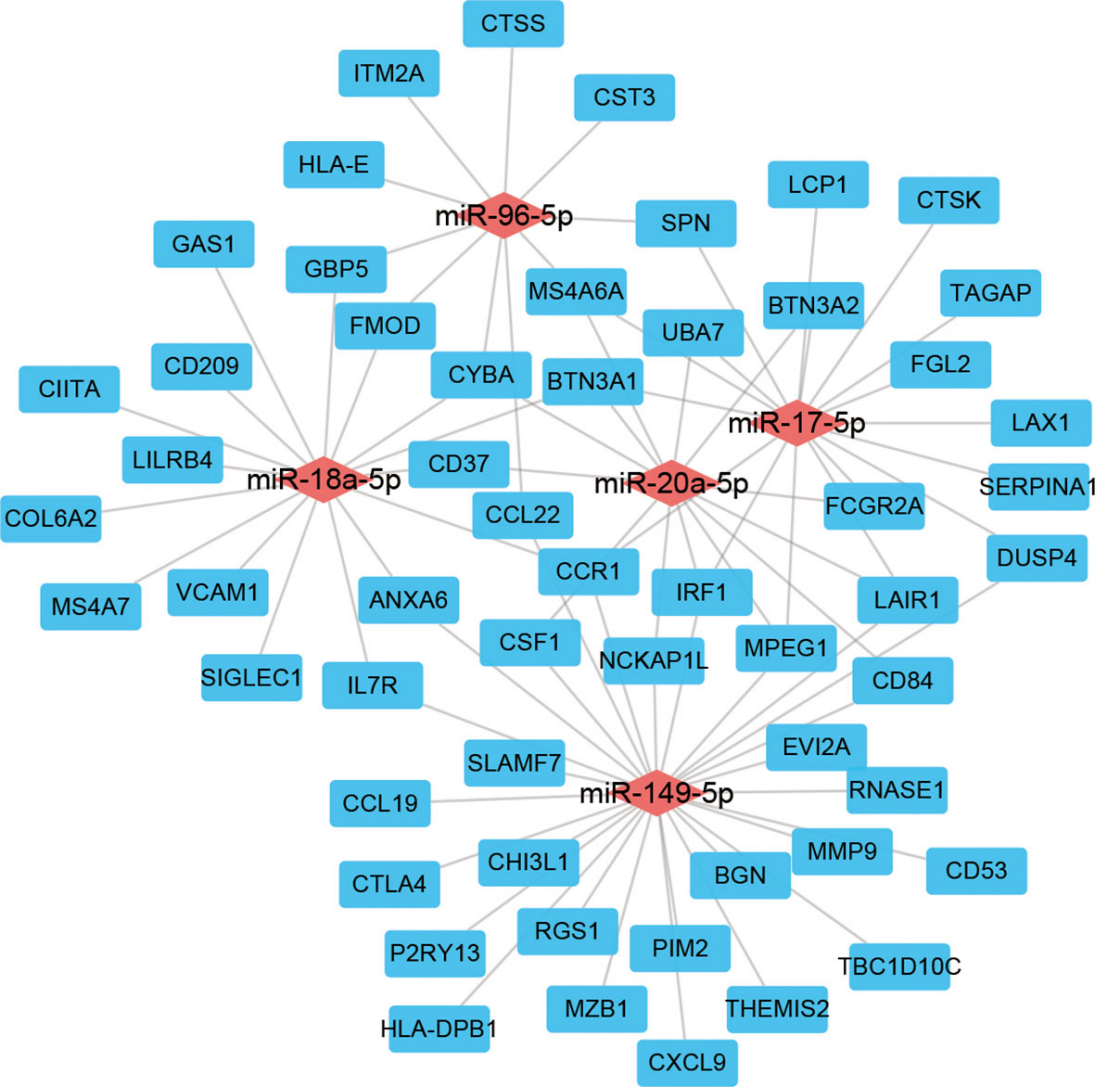
ceRNA network of genes, miRNAs, and lncRNAs differentially expressed both in the TCGA and GEO cohorts.

To investigate the regulatory network associated with immune infiltration phenotypes in LSCC patients, we constructed a ceRNA network using the genes, miRNAs, and lncRNAs those differentially expressed both in the TCGA and the GEO data (**Figure 5** and **Supplementary Table 2**). In this net, miR-149-5p regulated 28 DEGs while miR-17-5p, miR-18a-5p regulated 17 and 16 DEGs, respectively. Seven DEGs (BTN3A1, CCR1, CYBA, IRF1, LAIR1, MPEG1, and MS4A6A) were regulated by three miRNAs together.

### Construction of a Gene Expression Model to Predict the Immune Infiltration Phenotypes

In the above, we used GSVA and cluster analysis to determine the immune infiltration subtypes of the patients. However, this method is not suitable for analyzing the expression data of only one patient or quite a few patients because cluster analysis cannot be properly done at that time. To solve this problem, we constructed a predictive model using LASSO and binary logistic regression based on the expression of a set of genes to calculate which immune infiltration phenotype a patient belongs to.

**Figure 6:**
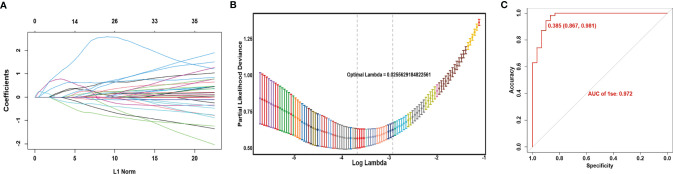
Construction of gene expression model to predict the immune infiltration phenotypes. **(A)** Coefficient profiles of variables in the LASSO regression model. **(B)** Tenfold cross-validation for turning parameter (λ) selection in the LASSO Cox regression model. **(C)** ROC curve of the model in the TCGA cohort.

Genes differentially expressed both in the TCGA and GEO cohorts were included in the LASSO analysis. The TCGA data were used as the training cohort while the GEO data were the validation cohort. Sixteen DEGs were selected in the logistic regression when lambda.min was chosen as the optimal parameter λ in the LASSO analysis (**Figures 6A**, **B**). Finally, a model was constructed as follows.


$$\begin{array}{c} \text{Score}=-9.11+(1*\text{IRF}4)+(-0.49*\text{SPN})+(5.4*\text{GIMAP}1)+(0.03*\text{MZB}1) \end{array}$$

Here the expression value of each gene in this score is log2(FPKM+1). Most of these genes were significantly correlated with survival (**Supplementary Figure 4**). Based on the ROC curve, the best cutoff value of the score was 0.541, with a 96.3% accuracy, 93.3% specificity, and 0.983 AUC (**Figure 6C**). When the gene expression data of an LSCC sample is substituted into the above formula, and the calculation result is less than 0.541, it indicates that the patient belongs to the high-infiltration phenotype. Otherwise, he belongs to the low-infiltration group.

We calculated the score’s performance in the GEO cohort, and found it also had an excellent performance with a 91.0% accuracy and 97.1% specificity, which demonstrated the universal usability of this score.

## Discussion

The immune infiltration phenotypes of LSCC have not been well analyzed before. In this study, we calculated the infiltration of each immunity cells in each LSCC patient of the TCGA database, and clustered the patients into high-infiltration and low-infiltration groups. Then we analyzed the differentially mutated genes, investigated and validated the differentially expressed genes, miRNAs, and lncRNAs between two immune infiltration groups. We also constructed a ceRNA network to explore the different molecular regulations and a predictive expression signature to determine a patient’s immune infiltration phenotype. The immune infiltration phenotype revealed in our study may act as immunotherapeutic biomarkers and potential therapeutic targets for LSCC.

Several studies have reported that the infiltration of specific immune cells correlated with the prognosis of LSCC. Both Chatzopoulos et al. (15) and Spector et al. (16) found the favorable prognostic impact of higher tumor-infiltrating lymphocytes in patients with LSCC. Spector et al. (16) also reported that low CD4 levels were associated with worse survival in the patients with chemoradiation more than those with surgery. Mann et al. (17) reported that high tumor CD103^+^ TIL content was associated with significantly improved survival in recurrent/persistent LSCC. Our study found significantly different enrichment statuses among immune cells such as CD8+ T cells and NK cells, which have been shown to enhance anti-tumor immunity and resulted in better survival in cancer patients (18).

We analyzed the differences in the mutation status between patients with high and low immune infiltration phenotypes, and found that COL11A1 and MUC17 were much more frequently mutated in the high-infiltration group. COL11A1 encodes a chain of type XI collagen, which locates in the extracellular matrix, and is up-regulated in various cancers (19). Song et al. (20) reported that COL11A1 more frequently mutated in head and neck cancer patients with high immunity, consistent with our results. Wu et al. (21) found that COL11A1 activated cancer-associated fibroblasts by modulating TGF-β3 through the NF-κB/IGFBP2 axis. As a membrane-bound mucin, MUC17 is instrumental in the trafficking and anchoring of receptor proteins and organizing signaling complexes at cellular membranes (22). However, the mutations of these genes have not been well investigated in LSCC, their roles in immune-infiltration still need to be further explored.

Immunotherapy is now developing rapidly and widely used in treating LSCC in combination with surgery, radiotherapy, and chemotherapy (23–26). Our results found that common targets of immunotherapies, such as PD1, PDL1, and CTAL-4, were all significantly up-regulated in patients with high-infiltration phenotype, suggesting that those patients might be more suitable to be immunotherapy. miR-149-5p, miR-17-5p, and miR-18a-5p were significantly down-regulated in the high-infiltration group, and acted as the hubs in the ceRNA network in our study. They are rarely studied in LSCC. Only Wang et al. (27) reported that miR-17-5p promoted proliferation and attenuated apoptosis *via* targeting PIK3R1 in LSCC, perhaps accounting for the poorer survival of the low-infiltration group in our study. The lncRNA, AL928768.3, up-regulated in the high-infiltration group both in the TCGA and GEO cohorts, its function in tumors is still not reported until now.

Finally, using LASSO regression, we constructed a model to calculate the immune infiltration phenotype of a patient based on the expression values of 16 genes. The LASSO regression method uses a penalty function to get a more refined regression model and reduce the overfitting, which is now widely used in genetic prediction (28, 29). Our model showed excellent accuracy and specificity in both the TCGA and GEO cohorts, suggesting it can be effectively used to classify the immune infiltration phenotypes of patients with LSCC and possibly predict the outcome of immunotherapy.

There are several limitations in our study. First, there are no public expression data of LSCC patients who received immunotherapy, so we can not validate our findings in patients who received immunotherapy. Meanwhile, it is worth noting that our model was only validated in 57 patients, with a relatively small number of samples and a lack of clinical validation. If the sample size in the validation cohort were larger, the results would be more convinced. We hope our results can be validated in larger cohorts in the future.

## Conclusions

In this study, we depicted the immune infiltration phenotypes of LSCC, and systemically analyzed the multi-omics differences between high-infiltration and low-infiltration groups. We also constructed an expression-based model to calculate a patient’s immune infiltration phenotype, which might be valuable to predicting immunotherapy’s outcome.

## Data Availability Statement

Data can be obtained from the UCSC Xena browser (https://gdc.xenahubs.net) and the GEO database (https://www.ncbi.nlm.nih.gov/gds/) under the accession numbers GSE127165 and GSE133632.

## Ethics Statement

The study was approved by the Ethics Committee of Eye & ENT Hospital, Fudan University (2020029-1).

## Author Contributions

LY, XS, GY, and LZ performed the research. LZ, YZ, and XW designed the research study. LY, LZ, YZ, and XW contributed essential reagents or tools. LY, XS, and GY analysed the data. LY, XS, and XW wrote the paper. All authors contributed to the article and approved the submitted version.

## Funding

This work was supported by the Foundation of Shanghai Municipal Commission of Health and Family Planning (20184Y0204).

## Conflict of Interest

The authors declare that the research was conducted in the absence of any commercial or financial relationships that could be construed as a potential conflict of interest.

## Publisher’s Note

All claims expressed in this article are solely those of the authors and do not necessarily represent those of their affiliated organizations, or those of the publisher, the editors and the reviewers. Any product that may be evaluated in this article, or claim that may be made by its manufacturer, is not guaranteed or endorsed by the publisher.

## Supplementary Material

Supplementary Figure 1 Mutation details of LSCC patients with different immune-infiltration phenotypes in the TCGA cohort. **(A)** Patients with high-infiltration phenotype. **(B)** Patients with low-infiltration phenotype.

Supplementary Figure 2 GO and KEGG analyses of DEGs in LSCC patients with different immune-infiltration phenotypes in the GEO cohort.

Supplementary Figure 3 Venn Plots of genes, miRNAs, and lncRNAs differentially expressed both in the TCGA and GEO cohorts. **(A)** genes. **(B)** miRNAs. **(C)** lncRNAs.

Supplementary Figure 4 Survival analyses of genes in the model.
