# Supplementary figures and images for: Identification and Validation of Immune Infiltration Phenotypes in Laryngeal Squamous Cell Carcinoma by Integrative Multi-Omics Analysis
Source: Front Immunol. 2022 Feb 24;13:843467. doi: 10.3389/fimmu.2022.843467 (PMC8907422; doi:10.3389/fimmu.2022.843467)

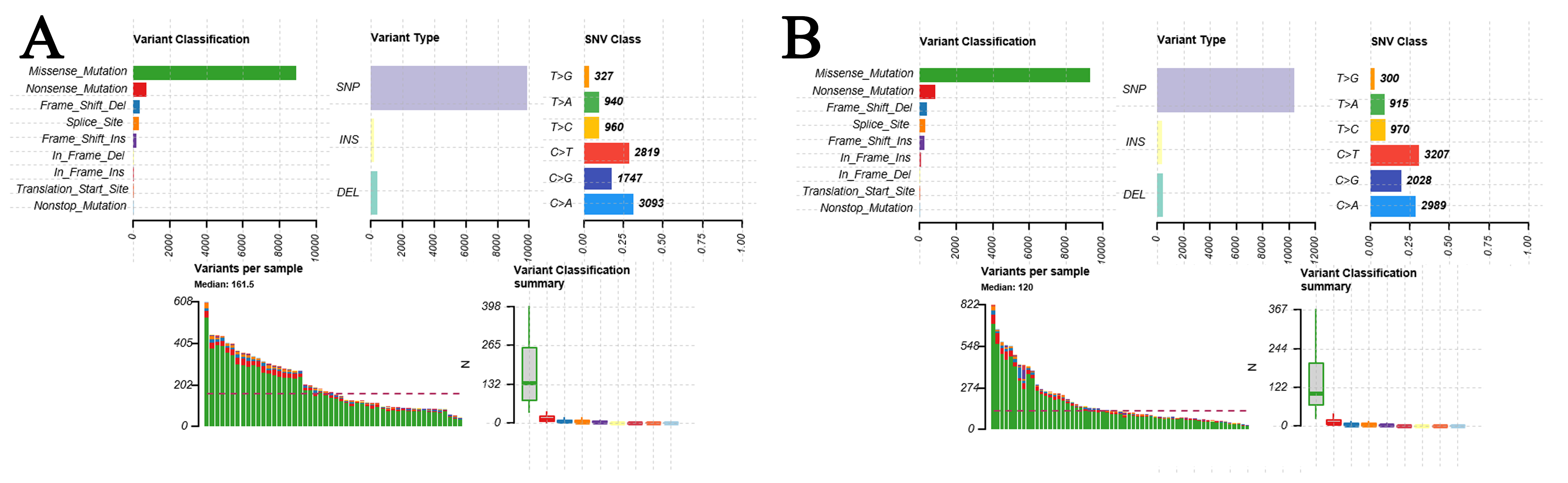

Supplement: Supplementary Figure 1 — Mutation details of LSCC patients with different immune-infiltration phenotypes in the TCGA cohort. (A) Patients with high-infiltration phenotype. (B) Patients with low-infiltration phenotype. [file Image_1.tif]

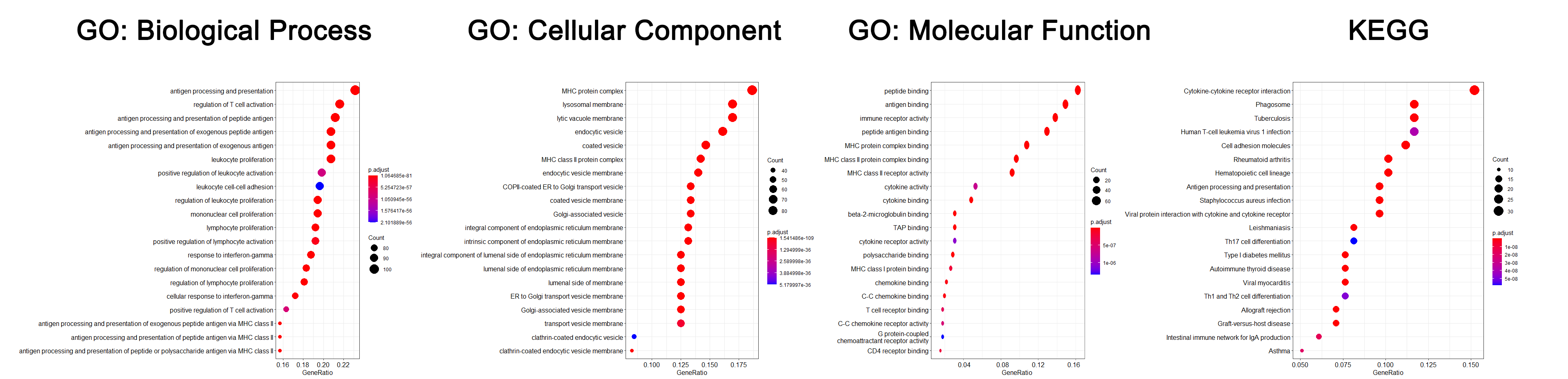

Supplement: Supplementary Figure 2 — GO and KEGG analyses of DEGs in LSCC patients with different immune-infiltration phenotypes in the GEO cohort. [file Image_2.tif]

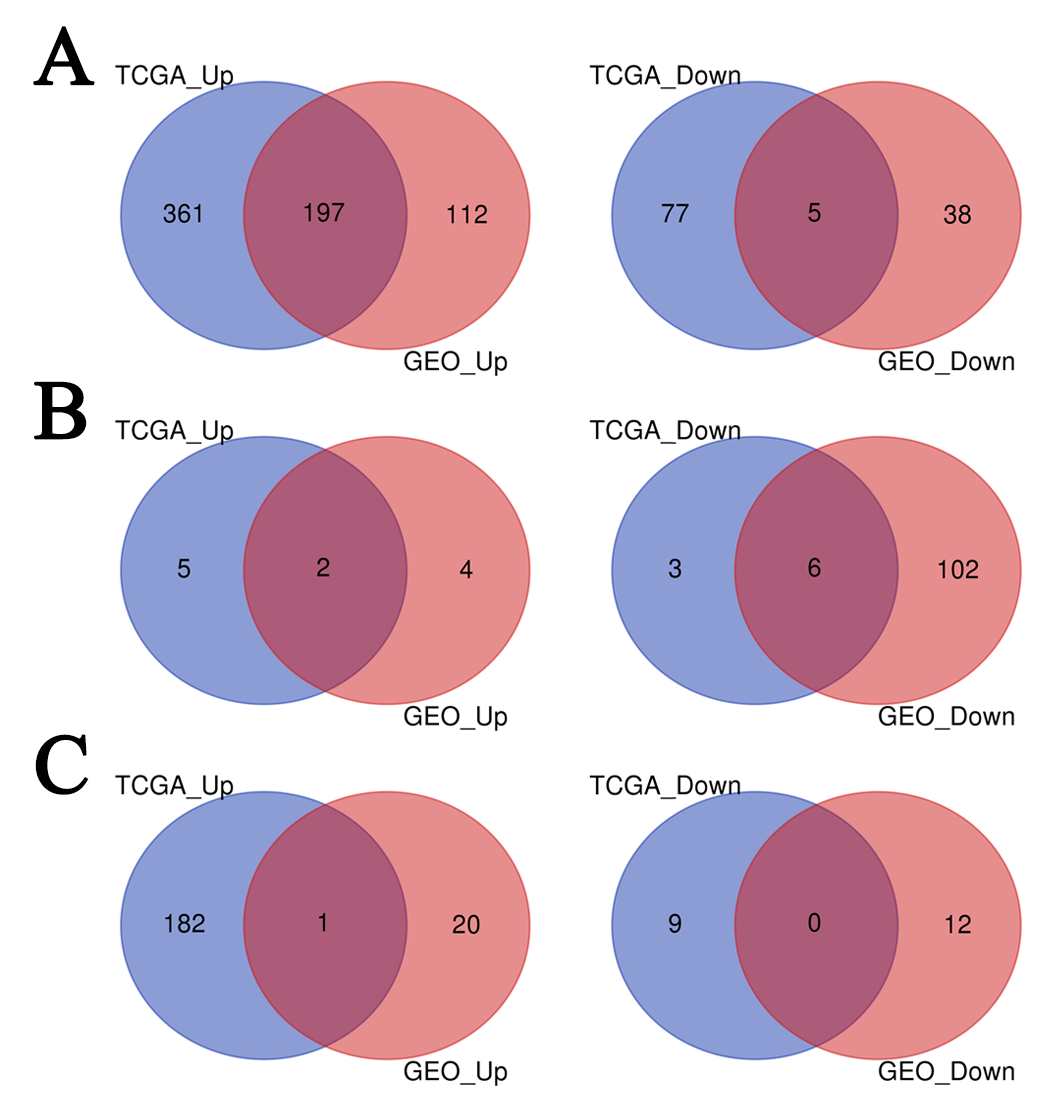

Supplement: Supplementary Figure 3 — Venn Plots of genes, miRNAs, and lncRNAs differentially expressed both in the TCGA and GEO cohorts. (A) genes. (B) miRNAs. (C) lncRNAs. [file Image_3.tif]

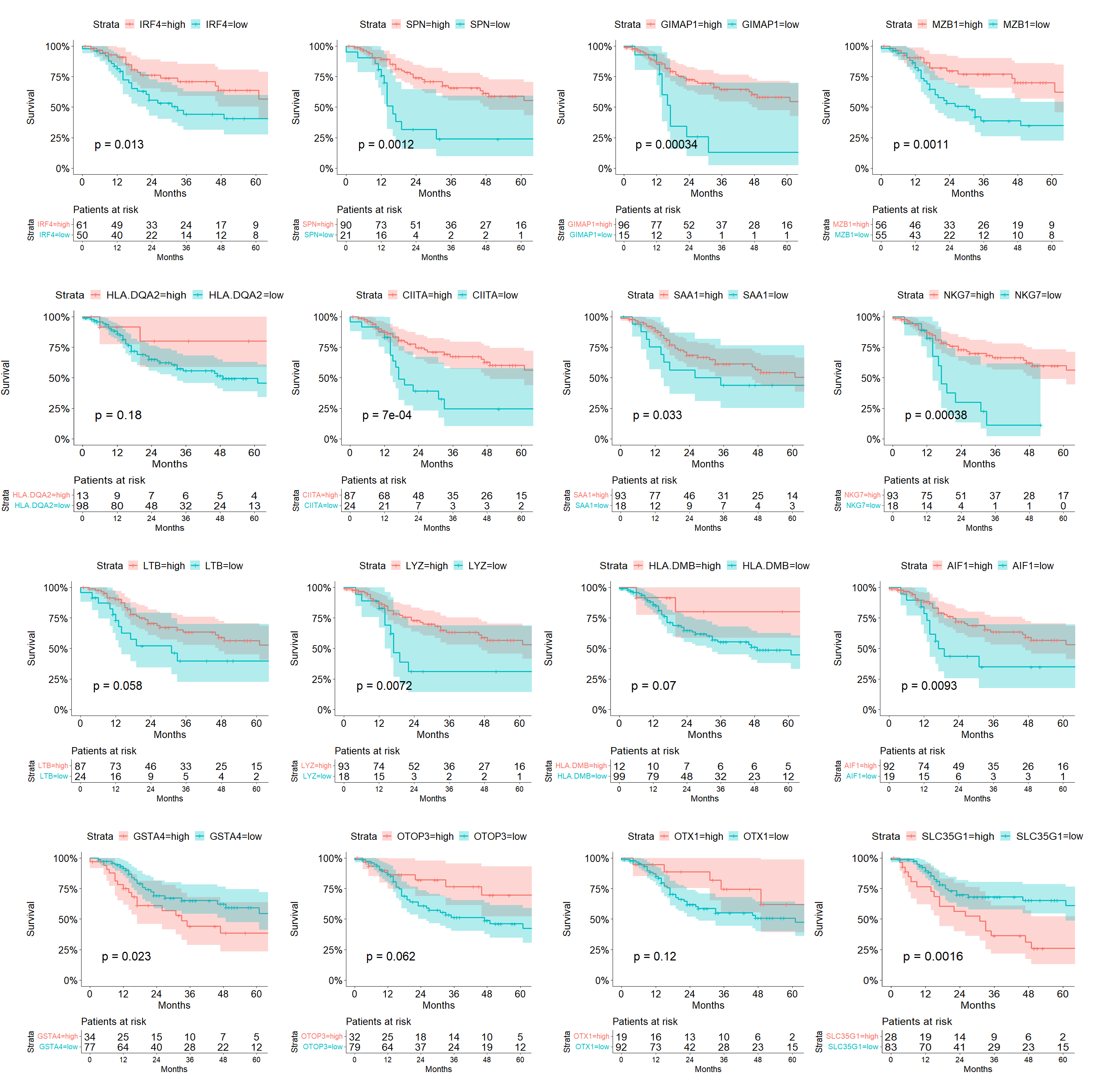

Supplement: Supplementary Figure 4 — Survival analyses of genes in the model. [file Image_4.tif]
