# Supplementary material for: Identification and Validation of Immune Infiltration Phenotypes in Laryngeal Squamous Cell Carcinoma by Integrative Multi-Omics Analysis
Source: Front Immunol. 2022 Feb 24;13:843467. doi: 10.3389/fimmu.2022.843467 (PMC8907422; doi:10.3389/fimmu.2022.843467)
Supplement: Supplementary file 5 [file Table_1.docx]

**Table S1.** Gene mutation status of LSCC patients with different immune infiltration phenotypes in the TCGA data cohort.

| Gene_Symbol | Low-Infiltration | High-Infiltration | p value |
| --- | --- | --- | --- |
| COL11A1 | 14 | 4 | 0.003944 |
| CDH9 | 11 | 2 | 0.005622 |
| LRRK2 | 0 | 8 | 0.00725 |
| LRP4 | 6 | 0 | 0.007847 |
| TEX13A | 6 | 0 | 0.007847 |
| REG1A | 8 | 1 | 0.010965 |
| GRIA3 | 5 | 0 | 0.018136 |
| JAG1 | 5 | 0 | 0.018136 |
| OR4A15 | 5 | 0 | 0.018136 |
| PCDHGA3 | 5 | 0 | 0.018136 |
| VCAM1 | 5 | 0 | 0.018136 |
| ZNF716 | 5 | 0 | 0.018136 |
| MUC17 | 13 | 5 | 0.019283 |
| DPYD | 1 | 9 | 0.019918 |
| GRM7 | 7 | 1 | 0.022762 |
| NCAM2 | 7 | 1 | 0.022762 |
| FAM135B | 15 | 7 | 0.029805 |
| MRC1 | 0 | 6 | 0.030098 |
| NRG3 | 1 | 8 | 0.03697 |
| ADCK4 | 4 | 0 | 0.041397 |
| CAMTA2 | 4 | 0 | 0.041397 |
| CCL1 | 4 | 0 | 0.041397 |
| CD1E | 4 | 0 | 0.041397 |
| CDK8 | 4 | 0 | 0.041397 |
| CEP250 | 4 | 0 | 0.041397 |
| CLSTN2 | 4 | 0 | 0.041397 |
| CNKSR2 | 4 | 0 | 0.041397 |
| CNTN5 | 4 | 0 | 0.041397 |
| COL15A1 | 4 | 0 | 0.041397 |
| DOPEY1 | 4 | 0 | 0.041397 |
| FAR1 | 4 | 0 | 0.041397 |
| FLNC | 4 | 0 | 0.041397 |
| GFRAL | 4 | 0 | 0.041397 |
| GRIA4 | 4 | 0 | 0.041397 |
| KIAA1257 | 4 | 0 | 0.041397 |
| KRT6A | 4 | 0 | 0.041397 |
| LHCGR | 4 | 0 | 0.041397 |
| MACROD2 | 4 | 0 | 0.041397 |
| NRCAM | 4 | 0 | 0.041397 |
| OR2F2 | 4 | 0 | 0.041397 |
| OR8D4 | 4 | 0 | 0.041397 |
| PCDHA2 | 4 | 0 | 0.041397 |
| PCDHGA12 | 4 | 0 | 0.041397 |
| PIP5K1B | 4 | 0 | 0.041397 |
| PTPN13 | 4 | 0 | 0.041397 |
| REV3L | 4 | 0 | 0.041397 |
| SERPINA9 | 4 | 0 | 0.041397 |
| STON1-GTF2A1L | 4 | 0 | 0.041397 |
| WSCD2 | 4 | 0 | 0.041397 |
| ZNF280B | 4 | 0 | 0.041397 |
| ZNF616 | 4 | 0 | 0.041397 |
| DCC | 8 | 2 | 0.041464 |
| DSP | 8 | 2 | 0.041464 |
| ZNF804A | 8 | 2 | 0.041464 |
| XIRP2 | 13 | 6 | 0.042056 |
| AFF2 | 6 | 1 | 0.046266 |
| ASTN2 | 6 | 1 | 0.046266 |
| MDGA2 | 6 | 1 | 0.046266 |
| FAT3 | 10 | 4 | 0.048064 |
