# Supplementary material for: Identification and Validation of Immune Infiltration Phenotypes in Laryngeal Squamous Cell Carcinoma by Integrative Multi-Omics Analysis
Source: Front Immunol. 2022 Feb 24;13:843467. doi: 10.3389/fimmu.2022.843467 (PMC8907422; doi:10.3389/fimmu.2022.843467)
Supplement: Supplementary file 6 [file Table_2.docx]

**Table S2.** Genes, miRNAs, and lncRNAs differentially expressed both in the TCGA and GEO cohorts.

| Type | Name | High-Infiltration/Low-Infiltration |
| --- | --- | --- |
| gene | IRF4 | up |
| gene | P2RY13 | up |
| gene | CXCR4 | up |
| gene | FGL2 | up |
| gene | HLA-F | up |
| gene | CYBA | up |
| gene | CD163 | up |
| gene | CD37 | up |
| gene | C1S | up |
| gene | C1R | up |
| gene | FCGR2A | up |
| gene | LILRB2 | up |
| gene | MFAP4 | up |
| gene | CECR1 | up |
| gene | C1QC | up |
| gene | TYROBP | up |
| gene | MS4A1 | up |
| gene | C16orf54 | up |
| gene | CD4 | up |
| gene | IRF1 | up |
| gene | SPN | up |
| gene | FPR3 | up |
| gene | PCOLCE | up |
| gene | RNASE6 | up |
| gene | CCR1 | up |
| gene | ARHGAP9 | up |
| gene | PTGDS | up |
| gene | LAX1 | up |
| gene | CCL22 | up |
| gene | HMHA1 | up |
| gene | HCST | up |
| gene | A2M | up |
| gene | ACP5 | up |
| gene | GPR183 | up |
| gene | APOBR | up |
| gene | RARRES2 | up |
| gene | RAC2 | up |
| gene | GZMA | up |
| gene | SPI1 | up |
| gene | ACKR1 | up |
| gene | CCL19 | up |
| gene | DUSP4 | up |
| gene | LAIR1 | up |
| gene | EVI2A | up |
| gene | CD79A | up |
| gene | GIMAP7 | up |
| gene | GIMAP1 | up |
| gene | CXCR3 | up |
| gene | APOE | up |
| gene | CTLA4 | up |
| gene | HLA-E | up |
| gene | SIRPA | up |
| gene | C3 | up |
| gene | BTN3A2 | up |
| gene | LILRB4 | up |
| gene | STAB1 | up |
| gene | UBA7 | up |
| gene | DOK3 | up |
| gene | BTN3A1 | up |
| gene | MZB1 | up |
| gene | GIMAP4 | up |
| gene | CSF1 | up |
| gene | MGAT1 | up |
| gene | SELPLG | up |
| gene | SERPING1 | up |
| gene | TBC1D10C | up |
| gene | HLA-DPB1 | up |
| gene | EMILIN1 | up |
| gene | SASH3 | up |
| gene | CD74 | up |
| gene | CORO1A | up |
| gene | HLA-DRA | up |
| gene | EVI2B | up |
| gene | GPSM3 | up |
| gene | CD52 | up |
| gene | NCKAP1L | up |
| gene | CXCL9 | up |
| gene | HLA-DQA2 | up |
| gene | TNFRSF1B | up |
| gene | AEBP1 | up |
| gene | GZMB | up |
| gene | CTSL | up |
| gene | CYBB | up |
| gene | FERMT3 | up |
| gene | CST3 | up |
| gene | CHI3L1 | up |
| gene | CD53 | up |
| gene | CIITA | up |
| gene | AKNA | up |
| gene | SERPINA1 | up |
| gene | C1QB | up |
| gene | TNFRSF14 | up |
| gene | SIGLEC10 | up |
| gene | SAA1 | up |
| gene | ITM2A | up |
| gene | SLAMF7 | up |
| gene | MS4A6A | up |
| gene | MAFB | up |
| gene | NKG7 | up |
| gene | TAGAP | up |
| gene | IL7R | up |
| gene | GAS1 | up |
| gene | CD209 | up |
| gene | MS4A7 | up |
| gene | CCL21 | up |
| gene | IL10RA | up |
| gene | RNASE1 | up |
| gene | ICAM3 | up |
| gene | TNFRSF4 | up |
| gene | VCAM1 | up |
| gene | ANXA6 | up |
| gene | PDGFRB | up |
| gene | CTSS | up |
| gene | COL6A2 | up |
| gene | HLA-DMA | up |
| gene | HLA-DPA1 | up |
| gene | HLA-DRB1 | up |
| gene | CCR2 | up |
| gene | IL32 | up |
| gene | VIM | up |
| gene | JAK3 | up |
| gene | LTB | up |
| gene | CD84 | up |
| gene | HLA-DOA | up |
| gene | LYZ | up |
| gene | HLA-DMB | up |
| gene | LAPTM5 | up |
| gene | OLFML3 | up |
| gene | CD7 | up |
| gene | CD27 | up |
| gene | LCP1 | up |
| gene | DOK2 | up |
| gene | TNFRSF17 | up |
| gene | MPEG1 | up |
| gene | CCR5 | up |
| gene | SLAMF8 | up |
| gene | MYO1G | up |
| gene | RASAL3 | up |
| gene | CD2 | up |
| gene | TMEM176A | up |
| gene | IRF8 | up |
| gene | IGLL5 | up |
| gene | FAM46C | up |
| gene | CD97 | up |
| gene | CXCR6 | up |
| gene | CTSK | up |
| gene | SIGLEC1 | up |
| gene | FCER1G | up |
| gene | PRF1 | up |
| gene | LST1 | up |
| gene | KCTD12 | up |
| gene | CD79B | up |
| gene | FMOD | up |
| gene | C1QA | up |
| gene | CTSZ | up |
| gene | PRELP | up |
| gene | SRGN | up |
| gene | TMEM119 | up |
| gene | HLA-C | up |
| gene | GBP5 | up |
| gene | ITGB2 | up |
| gene | FOLR2 | up |
| gene | SIT1 | up |
| gene | CD3D | up |
| gene | C10orf54 | up |
| gene | C10orf10 | up |
| gene | GIMAP6 | up |
| gene | UBD | up |
| gene | CLEC10A | up |
| gene | CSF1R | up |
| gene | AIF1 | up |
| gene | BGN | up |
| gene | THEMIS2 | up |
| gene | CD3E | up |
| gene | CCL2 | up |
| gene | PIM2 | up |
| gene | FAM26F | up |
| gene | C1orf162 | up |
| gene | TMEM176B | up |
| gene | CSF2RB | up |
| gene | IGJ | up |
| gene | ATP2A3 | up |
| gene | CCR7 | up |
| gene | RGS1 | up |
| gene | UCP2 | up |
| gene | GPR15 | up |
| gene | MMP9 | up |
| gene | FMNL1 | up |
| gene | ACAP1 | up |
| gene | CCR4 | up |
| gene | PTPRC | up |
| gene | GNLY | up |
| gene | IL2RG | up |
| gene | CCL13 | up |
| gene | DERL3 | up |
| gene | CD14 | up |
| gene | ARHGDIB | up |
| gene | GSTA4 | down |
| gene | OTOP3 | down |
| gene | OTX1 | down |
| gene | EPCAM | down |
| gene | SLC35G1 | down |
| miRNA | miR-96-5p | down |
| miRNA | miR-17-5p | down |
| miRNA | miR-19a-3p | down |
| miRNA | miR-18a-5p | down |
| miRNA | miR-20a-5p | down |
| miRNA | miR-149-5p | down |
| miRNA | miR-150-5p | up |
| miRNA | miR-146a-5p | up |
| lncRNA | AL928768.3 | up |
